# Diagnostic timeliness in adolescents and young adults with cancer: a cross-sectional analysis of the BRIGHTLIGHT cohort

**DOI:** 10.1016/S2352-4642(18)30004-X

**Published:** 2018-03

**Authors:** Annie Herbert, Georgios Lyratzopoulos, Jeremy Whelan, Rachel M Taylor, Julie Barber, Faith Gibson, Lorna A Fern

**Affiliations:** a Epidemiology of Cancer Healthcare and Outcomes (ECHO) Research Group, Department of Behavioural Sciences & Health, University College London, London, UK; b Department of Statistical Science, University College London, London, UK; c Cancer Division, University College London Hospitals NHS Foundation Trust, London, UK; d Centre for Outcomes and Experience Research in Children's Health, Illness and Disability (ORCHID), Great Ormond Street Hospital for Children NHS Foundation Trust, London, UK; e School of Health Sciences, University of Surrey, Guildford, UK

## Abstract

**Background:**

Adolescents and young adults (AYAs) are thought to experience prolonged intervals to cancer diagnosis, but evidence quantifying this hypothesis and identifying high-risk patient subgroups is insufficient. We aimed to investigate diagnostic timeliness in a cohort of AYAs with incident cancers and to identify factors associated with variation in timeliness.

**Methods:**

We did a cross-sectional analysis of the BRIGHTLIGHT cohort, which included AYAs aged 12–24 years recruited within an average of 6 months from new primary cancer diagnosis from 96 National Health Service hospitals across England between July 1, 2012, and April 30, 2015. Participants completed structured, face-to-face interviews to provide information on their diagnostic experience (eg, month and year of symptom onset, number of consultations before referral to specialist care); demographic information was extracted from case report forms and date of diagnosis and cancer type from the national cancer registry. We analysed these data to assess patient interval (time from symptom onset to first presentation to a general practitioner [GP] or emergency department), the number of prereferral GP consultations, and the symptom onset-to-diagnosis interval (time from symptom onset to diagnosis) by patient characteristic and cancer site, and examined associations using multivariable regression models.

**Findings:**

Of 1114 participants recruited to the BRIGHTLIGHT cohort, 830 completed a face-to-face interview. Among participants with available information, 204 (27%) of 748 had a patient interval of more than a month and 242 (35%) of 701 consulting a general practitioner had three or more prereferral consultations. The median symptom onset-to-diagnosis interval was 62 days (IQR 29–153). Compared with male AYAs, female AYAs were more likely to have three or more consultations (adjusted odds ratio [OR] 1·6 [95% CI 1·1–2·3], p=0·0093) and longer median symptom onset-to-diagnosis intervals (adjusted median interval longer by 24 days [95% CI 11–37], p=0·0005). Patients with lymphoma or bone tumours (adjusted OR 1·2 [95% CI 0·6–2·1] compared with lymphoma) were most likely to have three or more consultations and those with melanoma least likely (0·2 [0·1–0·7] compared with lymphoma). The adjusted median symptom onset-to-diagnosis intervals were longest in AYAs with bone tumours (51 days [95% CI 29–73] longer than for lymphoma) and shortest in those with leukaemia (33 days [17–49] shorter than for lymphoma).

**Interpretation:**

The findings provide a benchmark for diagnostic timeliness in young people with cancer and help to identify subgroups at higher risk of a prolonged diagnostic journey. Further research is needed to understand reasons for these findings and to prioritise and stratify early diagnosis initiatives for AYAs.

**Funding:**

National Institute for Health Research, Teenage Cancer Trust, and Cancer Research UK.

## Introduction

Cancer is the leading cause of disease-related death for adolescents and young adults (AYAs) in high-income countries.^1^ The lower age of AYAs is typically around 13 years but the upper range (of young adults) varies depending on jurisdiction from 24 years in the UK to 39 years in the USA.^1^ Improvements in survival for this population have lagged behind both children and older adults,^2^ probably as a result of distinct cancer and host biology, historically limited support for research combined with barriers to accessing existing studies, and prolonged pathways to diagnosis in this age group.^3^, ^4^

Most cancers are diagnosed after symptom onset.^5^ Some evidence suggests that AYAs experience a longer time from symptom onset to diagnosis than children and older adults.^4^, ^6^, ^7^, ^8^, ^9^ However, the evidence is difficult to interpret and inconclusive because comparable studies are rare. Prolonged intervals to diagnosis can adversely affect clinical outcomes, decrease confidence of patients and parents in their doctors, and are associated with a poor experience of subsequent cancer care.^10^, ^11^ Nonetheless, diagnostic timeliness in AYAs is not well quantified and the identification of high-risk groups remains elusive.

Contributing factors to prolonged diagnostic intervals are likely to be multifactorial.^12^ Young people have poor knowledge of AYA cancers and their potential symptoms.^13^ In primary care, cancer suspicion in AYAs is understandably low because cancer is rare in this age group and a general practitioner (GP) might see only one AYA with cancer in their career. This is further complicated because symptoms and signs can be attributed to other more common illnesses due to the low positive predictive value of alarm symptoms in young people.^14^ For example, the reported positive predictive value of neck lump or mass for lymphoma (0·5%) would translate to five lymphoma diagnoses for every 1000 AYAs consulting with this symptom.^14^ Early recognition of cancer is key to timely treatment and can improve psychosocial and clinical outcomes.^15^ Such improvements are crucial in view of the potential societal and economic consequences of premature morbidity and mortality in young people with cancer.

**Research in context**

**Evidence before this study**

We searched MEDLINE/PubMed for studies published in any language between database inception and Oct 30, 2017, investigating diagnostic intervals and prereferral consultations in adolescents and young adults (AYA) aged 13–24 years with cancer, using the search terms: “intervals”, “diagnosis”, “time to diagnosis”, “delay”, “cancer”, “adolescents”, “teenagers”, and “young adults”. Additionally, we searched for papers by researchers and institutions that we knew had published in the field. We identified a systematic review on diagnostic intervals in children with cancer, and a review on diagnostic intervals in both children and young adults with cancer, including 32 studies reporting different measures or markers of diagnostic timeliness. The review in children and young adults included a single-institution, US study reporting total diagnostic intervals (from presentation to diagnosis) in patients aged 15–29 years with selected cancer types. We also identified a scoping review and five relevant studies: two studies used routine administrative data to detail primary-care use in young people before diagnosis, and the remaining three were single-centre studies that collected data through internal medical record review. Overall, evidence regarding diagnostic timeliness for AYAs is insufficient and heterogeneous because young people aged 13–24 years were usually included in studies of children (in which most were younger than 15 years) or older adults (in which most were aged 50 years or older). Furthermore, the range of cancer types occurring in AYAs is distinct from those found in children and older adults, and thus the data reported are chiefly related to cancer types that are less relevant to AYAs. Additionally, because of the unique nature of health-care use and the range of disease occurring in AYAs, extrapolations from data on children or older adults must be viewed with caution.

**Added value of this study**

To our knowledge, our study is the first to solely focus on adolescents and young adults aged 13–24 years with any cancer site, and our cohort is currently the largest of AYA patients with cancer with both patient-reported data and clinical data. All participants were recruited from a range of hospitals (some of which were AYA Principal Treatment Centres) and interviews were done within an average of 6 months of diagnosis, limiting the potential for recall bias. Furthermore, for the first time we examined variation in diagnostic timeliness by sociodemographic characteristic and identify subgroups at increased risk of prolonged diagnostic intervals. In particular, AYA patients with melanoma are most likely to have long patient intervals (time from symptom onset to first health-care presentation), and female AYAs are more likely to have multiple general practitioner consultations before referral to a cancer specialist than male AYAs; AYA patients with lymphoma or bone cancer (both common cancers in adolescence) are also more likely to have multiple general practitioner consultations before referral than AYA patients with other cancers.

**Implications of all the available evidence**

These results provide evidence for policy makers, clinicians, and researchers about subgroups of AYAs at increased risk of prolonged diagnostic experiences, either before or after presentation. The results therefore enable future targeting of public health education or health-care interventions, and can be used to guide the development of new diagnostic care services and diagnostic technology innovations for AYA patients with new symptoms. We also show the feasibility of studying diagnostic timeliness in this age group on the basis of self-reports, which might enable routine future repeat surveys internationally.

We aimed to investigate diagnostic timeliness of cancer in a cohort of young people to identify factors associated with prolonged diagnostic journeys and generate evidence to inform interventions supporting earlier diagnosis and improvement of subsequent outcomes.

## Methods

### Study design and participants

We did a cross-sectional analysis of the BRIGHTLIGHT cohort. BRIGHTLIGHT is a programme of research assessing specialist care for young people with cancer in England.^16^ This project involves data derived from patient reports enriched by patient-level information from case report forms completed by recruiting National Health Service (NHS) clinical treatment teams in addition to diagnosis (cancer site) and diagnosis date data by the National Cancer Registration and Analysis Service of Public Health England.

The BRIGHTLIGHT programme recruited participants within a few months of cancer diagnosis from 96 NHS Trust hospitals across England, which deliver free universal health care to all patients in their geographical catchment. Treatment teams identified and recruited patients aged 13–24 years with any new primary cancer diagnosis between July 1, 2012, and April 30, 2015. Patients excluded were those unable to complete the survey, unable to give consent, or facing imminent death. Young people serving a custodial sentence were also excluded due to the impracticalities of obtaining consent.

BRIGHTLIGHT was approved by London-Bloomsbury Research Ethics Committee (reference 11/LO/1718). In addition to their survey data, young people also gave written informed consent for clinical information to be extracted from their medical records; additional data were obtained from the Office for Data Release at Public Health England following Section 251 approval from the Confidentiality Advisory Group (reference ECC 8-05(d)/2011).

### Procedures

Young people's experience of cancer diagnosis was captured through structured, face-to-face interviews. Survey instrument development has been previously described.^17^ The overall survey instrument consists of 15 domains identified by young people during BRIGHTLIGHT feasibility work as important in their cancer care experience, including experience before and during diagnosis.^17^ The survey provider (Ipsos MORI) contacted participants to do the interview in a location of the patient's preference, mainly in their home. Questions were read out by the interviewer and answered from a list, with options for free text and for relevant dates. Patients answered questions about diagnostic events and intervals between their reported symptom onset and cancer diagnosis.

Sex, age at diagnosis, residential postcode (matched to Local Super Output Area and used to derive Index of Multiple Deprivation 2015 scores), and self-reported ethnicity were extracted from case report forms. Information on date of diagnosis and cancer type was extracted from the national cancer registry.

### Outcomes and exposure variables

**Figure 1:**
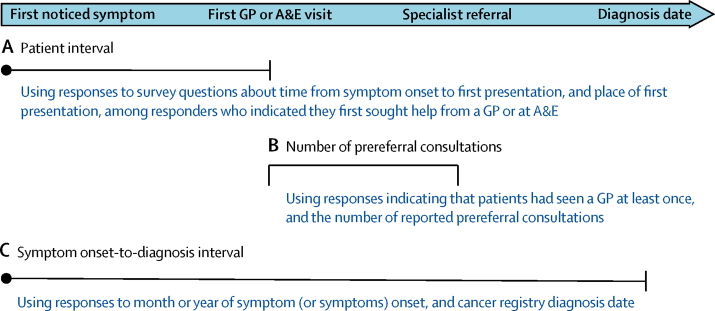
Outcome definitions and their relation to key aspects of the diagnostic process Relevant survey items and data sources used to define each outcome (A–C) are detailed in the appendix. A&E=accident and emergency department. GP=general practitioner.

We used five survey questions and diagnosis date from the cancer registry to define two interval measures and a proxy marker of diagnostic timeliness (figure 1; appendix) in accordance with an international consensus statement and previous work on related measures: the patient interval (from symptom onset to first presentation to a GP or the emergency department), three or more GP consultations before referral to specialist services (restricted to patients who reported to have consulted with a GP), and the symptom onset-to-diagnosis interval.^18^, ^19^, ^20^

Exposure variables used in the analysis were sex, age at diagnosis (grouped as 12–15 years, 16–18 years, and 19–24 years on the basis of recognised groupings of children and younger adolescents *vs* adolescents *vs* young adults),^21^ deprivation (grouped according to Index of Multiple Deprivation quintiles), ethnicity (white, black, Chinese, Asian, mixed, and other; grouped as white *vs* non-white), marital status (married or civil partnership, cohabiting, single, and divorced; grouped as married, civil partnership, or cohabiting *vs* single or divorced), and employment status (education, working full-time or part-time, other work [apprentice, internship, or voluntary], not seeking work, unemployed, and long-term sick). These exposure variables were selected because of their likely association with diagnostic timeliness as previously described by Walter and colleagues.^22^

We used Birch's morphology-based classification for AYA cancers, which is based on the International Classification of Diseases, to better reflect cancer incidence patterns in young people.^23^ Groups comprised leukaemia; lymphoma; CNS and other intracranial and intraspinal neoplasms (CNS); osseous and chondromatous neoplasms, Ewing's tumour, and other neoplasms of bone (bone tumours); soft tissue sarcomas; germ-cell and trophoblastic neoplasms (germ-cell tumours); melanoma and skin carcinoma; carcinomas (except of skin); and miscellaneous specified neoplasms not elsewhere classified and unspecified malignant neoplasms not elsewhere classified (grouped as unclassified or unspecified).

### Statistical analyses

Because this study comprised secondary data analyses, no sample size calculations were done. We calculated descriptive statistics for each outcome, overall and by exposure variable. For patient interval, we reported numbers and proportions of patients for different binary interval cutoffs. We also described the numbers and proportions of patients with three or more prereferral GP consultations as it is associated with the length of primary care interval.^19^ Because the symptom onset-to-diagnosis interval was a continuous variable (number of days), we reported descriptive statistics as 10th, 25th, 50th, 75th, and 90th centiles.

We additionally fitted multivariable regression models including all exposures as independent variables to account for potential confounding of crude associations by other variables, including all patients with complete information on outcome and exposure variables. The reference groups in these models were male; aged 12–15 years; least deprived; white; lymphoma (as the largest group by cancer site); married, in a civil partnership, or cohabiting; and in education. We tested for variation across multivariable categorical variables using joint Wald tests. We did not study interactions between exposures because we deemed the sample size was not large enough to enable such informative analysis.

For the two outcomes that were parameterised as categorical variables (ie, patient interval and prereferral GP consultations), we fitted multivariable logistic regression models. We categorised the patient interval as a binary variable (≤1 month *vs* >1 month), informed by previous relevant studies in adult patients.^24^, ^25^ We opted for a 1 month cutoff, deeming this long enough to be clinically important, and taking into consideration that public health education campaigns about awareness of cancer symptoms typically use a cutoff of 3 weeks or longer for duration of new symptoms. We treated the number of prereferral consultations as a binary outcome (<3 *vs* ≥3), consistent with public reporting conventions of the National Cancer Patient Experience Survey,^26^ and because some second appointments could reflect the need to review findings of investigations ordered at initial consultation.^27^

For the continuous outcome (symptom onset-to-diagnosis interval), we fitted multivariable quantile regression models with robust SEs.^28^ Quantile regression allows modelling at different quantiles of the outcome where associations between exposure and outcome can differ; we chose this modelling approach given statistical evidence for heterogeneity of associations for cancer site (eg, for soft tissue sarcoma p=0·01) and employment status (eg, for long-term sick p=0·04) in our sample. Because the univariate analysis did not show any clinically important variation at shorter durations of the symptom onset-to-diagnosis interval (eg, at the 10th centile), we fitted quantile regression to the 90th centile and the median symptom onset-to-diagnosis interval (ie, the 50th centile).

We analysed data in Stata, version 13.

### Role of the funding source

The funders of the study had no role in study design, data collection, data analysis, data interpretation, or writing of the report. The corresponding author had full access to all the data in the study and had final responsibility for the decision to submit for publication.

## Results

Among 1114 participants recruited to the BRIGHTLIGHT cohort, 830 completed a face-to-face interview. Reasons for dropout between giving consent and interview included early death, refusal, and illness; these participants were not atypical to those who remained in the study (appendix). The median age of participants interviewed was 20 years (IQR 17–22), 453 (55%) were male, and most were white (table 1). Five patients aged 12 years were recruited, because of discrepancies in dates of diagnosis between the recruiting centre and the cancer registry. We retained these patients in the study because they were very close to their 13th birthday at recruitment. Common cancer diagnoses were lymphoma, germ-cell tumours, leukaemia, carcinomas, and bone cancer (table 1). Information was complete for sex, age, and ethnicity, with low levels of missingness for other variables. The median time between diagnosis and interview was 185 days (IQR 161–220), and we did not find an association between the length of diagnosis-to-interview period and the three diagnostic timeliness measures assessed (appendix). The sociodemographic characteristics and cancer sites of BRIGHTLIGHT participants were overall similar to incident AYA cancer cases. A full description of the BRIGHTLIGHT cohort will be reported separately.

**Table 1:** Sociodemographic variables and cancer site

	Participants (n=830)*
**Sex**	
Male	453 (55%)
Female	377 (45%)
**Age at diagnosis (years)**	
Median (IQR)	20 (17–22)
12–15†	115 (14%)
16–18	187 (23%)
19–24	528 (64%)
**Index of Multiple Deprivation**	
First quintile (least deprived)	184 (22%)
Second quintile	136 (16%)
Third quintile	156 (19%)
Fourth quintile	182 (22%)
Fifth quintile (most deprived)	158 (19%)
Missing	14 (2%)
**Ethnic group**‡	
White	730 (88%)
Black	15 (2%)
Asian	61 (7%)
Chinese	4 (<1%)
Mixed	14 (2%)
Other	6 (1%)
**Cancer site**§**(descending order of most common)**	
Lymphoma	266 (32%)
Germ-cell tumours¶	156 (19%)
Leukaemia	105 (13%)
Carcinomas (non-skin)	100 (12%)
Bone cancer‖	79 (10%)
Soft tissue sarcomas	50 (6%)
CNS**	33 (4%)
Melanoma and skin	31 (4%)
Unclassified or unspecified	10 (1%)
**Marital status**††	
Married, civil partnership, or cohabiting	119 (14%)
Single or divorced	709 (85%)
Missing	2 (<1%)
**Employment status**‡‡	
Education	274 (33%)
Working full or part time	257 (31%)
Other work (apprentice, internship, or voluntary)	17 (2%)
Not seeking work	125 (15%)
Unemployed	31 (4%)
Long-term sick	126 (15%)

Data are n (%), unless stated otherwise. Missing category proportions only shown where applicable.

* Due to rounding, percentages do not always total 100%.

† Five patients were 12 years old.

‡ For analytical purposes, ethnicity was subsequently grouped as white versus non-white.

§ For the 70 (8%) patients for whom it was not possible to link data to the cancer registry, cancer site was taken from the participant registration form and checked against the case report form submitted by the clinical team.

¶ Germ-cell and trophoblastic neoplasms.

‖ Osseous and chondromatous neoplasms, Ewing's tumour, and other neoplasms of bone.

** CNS and other intracranial and intraspinal neoplasms.

†† 103 patients who were younger than 16 years and missing a response were assumed to be single.

‡‡ Employment status is based on response to the question “can you tell me what you're doing at the moment?”, and on a selection of multiple choice answers in the BRIGHTLIGHT survey. There were 13 possible responses, grouped into these six categories. For the 57 (7%) patients who gave more than one response, the category for analysis was selected by discussion (RMT and JB) on the basis of hierarchical rules.

**Figure 2:**
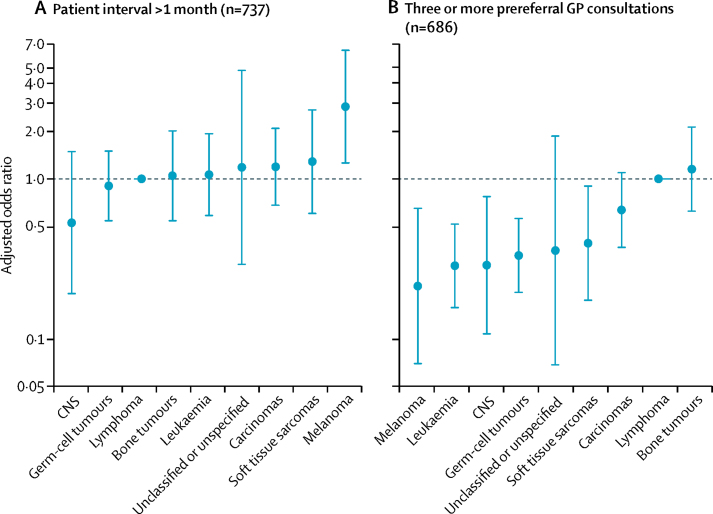
Adjusted odds ratios of (A) patient interval >1 month and (B) three or more prereferral GP consultations, by cancer site Error bars denote 95% CI. GP=general practitioner. Bone tumours=osseous and chondromatous neoplasms, Ewing's tumour, and other neoplasms of bone. CNS=CNS and other intracranial and intraspinal neoplasms. Germ-cell tumours=germ-cell and trophoblastic neoplasms.

754 of 830 participants reported first seeking help from a GP or an accident and emergency department; of these, six were excluded from analysis of patient interval because of missing outcome information. Among the remaining 748 patients, about half had an interval of more than 2 weeks, with about one-third having intervals of more than a month, and even fewer more than 3 months (table 2). Older patients (aged 19–24 years) or patients with melanoma had higher observed proportions of prolonged patient intervals than other patient groups (table 2). In multivariable regression analysis, the greatest degree of variation in the proportion of patients with a patient interval duration of more than a month was observed for cancer site and employment status, although without statistical evidence of significance. Compared with patients with lymphoma, those with melanoma were most likely to have a patient interval of more than a month (table 2; figure 2). About half of the patients in the other work (apprentice, internship, or voluntary) category had a patient interval of more than a month, although this estimate is based on small numbers (table 2).

**Table 2:** Associations of patient interval with sociodemographic variables and cancer site

		**All patients with complete patient interval data**				Regression analysis of patient interval >1 month*			
		N	>2 weeks	>1 month	>3 months	N	Crude percentage	Crude OR (95% CI)	Adjusted OR (95% CI)
All patients		748	359 (48%)	204 (27%)	91 (12%)	737	204 (28%)	NA	NA
Sex									
	Male	419	195 (47%)	107 (26%)	48 (11%)	414	107 (26%)	1 (ref)	1 (ref)
	Female	329	164 (50%)	97 (29%)	43 (13%)	323	97 (30%)	1·2 (0·9–1·7)	1·1 (0·8–1·6)
	p value	..	..	..	..	..	..	..	0·57
Age at diagnosis (years)									
	12–15	104	42 (40%)	22 (21%)	6 (6%)	100	22 (22%)	1 (ref)	1 (ref)
	16–18	170	83 (49%)	46 (27%)	18 (11%)	169	46 (27%)	1·3 (0·7–2·3)	1·3 (0·7–2·5)
	19–24	474	234 (49%)	136 (29%)	67 (14%)	468	136 (29%)	1·5 (0·9–2·5)	1·6 (0·8–3·1)
	p value	..	..	..	..	..	..	..	0·38
Index of Multiple Deprivation									
	First quintile (least deprived)	168	86 (51%)	49 (29%)	24 (14%)	168	49 (29%)	1 (ref)	1 (ref)
	Second quintile	121	53 (44%)	36 (30%)	15 (12%)	121	36 (30%)	1·0 (0·6–1·7)	1·1 (0·7–1·9)
	Third quintile	139	66 (47%)	40 (29%)	22 (16%)	139	40 (29%)	1·0 (0·6–1·6)	1·0 (0·6–1·7)
	Fourth quintile	164	88 (54%)	44 (27%)	13 (8%)	164	44 (27%)	0·9 (0·6–1·5)	0·9 (0·5–1·5)
	Fifth quintile (most deprived)	145	62 (43%)	35 (24%)	17 (12%)	145	35 (24%)	0·8 (0·5–1·3)	0·8 (0·5–1·4)
	p value	..	..	..	..	..	..	..	0·83
Ethnic group									
	White	657	321 (49%)	182 (28%)	84 (13%)	647	182 (28%)	1 (ref)	1 (ref)
	Non-white	91	38 (42%)	22 (24%)	7 (8%)	90	22 (24%)	0·8 (0·5–1·3)	0·8 (0·5–1·4)
	p value	..	..	..	..	..	..	..	0·44
Cancer site									
	Lymphoma	248	125 (50%)	66 (27%)	33 (13%)	247	66 (27%)	1 (ref)	1 (ref)
	Germ-cell tumours	147	66 (45%)	36 (24%)	17 (12%)	147	36 (24%)	0·9 (0·6–1·4)	0·9 (0·5–1·5)
	Leukaemia	89	42 (47%)	22 (25%)	5 (6%)	84	22 (26%)	1·0 (0·6–1·8)	1·1 (0·6–1·9)
	Carcinomas	87	43 (49%)	28 (32%)	13 (15%)	86	28 (33%)	1·3 (0·8–2·2)	1·2 (0·7–2·1)
	Bone tumours	68	33 (49%)	17 (25%)	6 (9%)	65	17 (26%)	1·0 (0·5–1·9)	1·0 (0·5–2·0)
	Soft tissue sarcomas	41	17 (41%)	13 (32%)	4 (10%)	40	13 (33%)	1·3 (0·6–2·7)	1·3 (0·6–2·7)
	CNS	30	10 (33%)	5 (17%)	2 (7%)	30	5 (17%)	0·5 (0·2–1·4)	0·5 (0·2–1·5)
	Melanoma	28	19 (68%)	14 (50%)	10 (36%)	28	14 (50%)	2·7 (1·2–6·0)	2·8 (1·3–6·4)
	Unclassified or unspecified	10	4 (40%)	3 (30%)	1 (10%)	10	3 (30%)	1·2 (0·3–4·8)	1·2 (0·3–4·8)
	p value	..	..	..	..	..	..	..	0·30
Marital status									
	Married, civil partnership, or cohabiting	106	56 (53%)	35 (33%)	14 (13%)	105	35 (33%)	1 (ref)	1 (ref)
	Single or divorced	642	303 (47%)	169 (26%)	77 (12%)	632	169 (27%)	0·7 (0·4–1·1)	0·8 (0·5–1·3)
	p value	..	..	..	..	..	..	..	0·39
Employment status									
	Education	249	115 (46%)	65 (26%)	28 (11%)	245	65 (27%)	1 (ref)	1 (ref)
	Working full-time or part-time	231	112 (48%)	61 (26%)	29 (13%)	229	61 (27%)	1·0 (0·7–1·5)	0·7 (0·4–1·2)
	Other work (apprentice, internship, or voluntary)	17	12 (71%)	8 (47%)	1 (6%)	16	8 (50%)	2·8 (1·0–7·8)	2·5 (0·9–7·1)
	Not seeking work	107	56 (52%)	28 (26%)	13 (12%)	105	28 (27%)	1·0 (0·6–1·7)	0·9 (0·5–1·5)
	Unemployed	28	9 (32%)	6 (21%)	4 (14%)	28	6 (21%)	0·8 (0·3–2·1)	0·6 (0·2–1·7)
	Long-term sick	116	55 (47%)	36 (31%)	16 (14%)	114	36 (32%)	1·3 (0·8–2·1)	1·0 (0·5–1·8)
	p value	..	..	..	..	..	..	..	0·22

Data are n (%) unless stated otherwise. Patient interval defined as time from first symptom to presentation to a general practitioner or accident and emergency department. OR=odds ratio. NA=not applicable.

* Patients with patient interval and covariate information.

Of 830 participants, 125 did not indicate that they had visited their GP prereferral, and four did not know their number of prereferral GP visits. After excluding these patients, 242 (35%) of 701 patients reported that they visited a GP three or more times (table 3). Larger proportions of patients who were female, white, diagnosed with lymphoma or bone tumours, or not seeking work or registered as long-term sick at the time of interview had three or more prereferral consultations (table 3). Among those aged 16–18 years, the observed proportion who visited a GP three or more times was slightly lower than that in younger or older AYAs. In multivariable analysis, there was significant variation in the proportion of patients with three or more consultations by sex and cancer site (table 3). Specifically, female AYAs were more likely to have three or more consultations than male AYAs, as were AYAs diagnosed with lymphoma or bone tumours compared with those with other cancers (table 3; figure 2). The cancer sites least associated with three or more prereferral consultations were melanoma, germ-cell tumours, and leukaemia (table 3; figure 2).

**Table 3:** Associations of three or more prereferral GP consultations with sociodemographic variables and cancer site*

		**Descriptive statistics for all patients with complete outcome information**		**Regression analysis of three or more prereferral GP consultations**†			
		N	n (%)	N	Crude percentage	Crude OR (95% CI)	Adjusted OR (95% CI)
All patients		701	242 (35%)	686	237 (35%)	NA	NA
Sex							
	Male	387	110 (28%)	378	106 (28%)	1 (ref)	1 (ref)
	Female	314	132 (42%)	308	131 (43%)	1·9 (1·4–2·6)	1·6 (1·1–2·3)
	p value	..	..	..	..	..	0·0093
Age at diagnosis (years)							
	12–15	93	35 (38%)	89	34 (38%)	1 (ref)	1 (ref)
	16–18	163	50 (31%)	160	49 (31%)	0·7 (0·4–1·2)	0·6 (0·4–1·2)
	19–24	445	157 (35%)	437	154 (35%)	0·9 (0·6–1·4)	0·8 (0·4–1·4)
	p value	..	..	..	..	..	0·36
Index of Multiple Deprivation							
	First quintile (least deprived)	157	51 (32%)	157	51 (32%)	1 (ref)	1 (ref)
	Second quintile	116	38 (33%)	114	38 (33%)	1·0 (0·6–1·7)	1·1 (0·7–2·0)
	Third quintile	137	51 (37%)	137	51 (37%)	1·2 (0·7–1·9)	1·3 (0·8–2·2)
	Fourth quintile	148	53 (36%)	148	53 (36%)	1·2 (0·7–1·9)	1·2 (0·7–2·0)
	Fifth quintile (most deprived)	130	44 (34%)	130	44 (34%)	1·1 (0·7–1·8)	1·0 (0·6–1·8)
	p value	..	..	..	..	..	0·85
Ethnic group							
	White	618	218 (35%)	604	214 (35%)	1 (ref)	1 (ref)
	Non-white	83	24 (29%)	82	23 (28%)	0·7 (0·4–1·2)	0·7 (0·4–1·2)
	p value	..	..	..	..	..	0·21
Cancer site							
	Lymphoma	231	108 (47%)	229	108 (47%)	1 (ref)	1 (ref)
	Germ-cell tumours	131	26 (20%)	131	26 (20%)	0·3 (0·2–0·5)	0·3 (0·2–0·6)
	Leukaemia	95	21 (22%)	89	19 (21%)	0·3 (0·2–0·5)	0·3 (0·2–0·5)
	Carcinomas	84	33 (39%)	83	33 (40%)	0·7 (0·4–1·2)	0·6 (0·4–1·1)
	Bone tumours	63	32 (51%)	60	30 (50%)	1·1 (0·6–1·9)	1·2 (0·6–2·1)
	Soft tissue sarcomas	39	10 (26%)	36	9 (25%)	0·4 (0·2–0·9)	0·4 (0·2–0·9)
	CNS	25	6 (24%)	25	6 (24%)	0·4 (0·2–1·0)	0·3 (0·1–0·8)
	Melanoma	25	4 (16%)	25	4 (16%)	0·2 (0·1–0·6)	0·2 (0·1–0·7)
	Unclassified or unspecified	8	2 (25%)	8	2 (25%)	0·4 (0·1–2·0)	0·4 (0·1–1·9)
	p value	..	..	..	..	..	<0·0001
Marital status							
	Married, civil partnership, or cohabiting	97	33 (34%)	96	33 (34%)	1 (ref)	1 (ref)
	Single or divorced	602	209 (35%)	590	204 (35%)	1·0 (0·6–1·6)	1·0 (0·6–1·7)
	p value	..	..	..	..	..	0·94
Employment status							
	Education	231	74 (32%)	225	72 (32%)	1 (ref)	1 (ref)
	Working full-time or part-time	221	71 (32%)	218	70 (32%)	1·0 (0·7–1·5)	1·1 (0·7–1·9)
	Other work (apprentice, internship, or voluntary)	15	5 (33%)	14	5 (36%)	1·2 (0·4–3·7)	1·2 (0·4–4·0)
	Not seeking work	103	39 (38%)	101	38 (38%)	1·3 (0·8–2·1)	1·1 (0·6–1·9)
	Unemployed	27	7 (26%)	27	7 (26%)	0·7 (0·3–1·7)	1·1 (0·4–3·0)
	Long-term sick	104	46 (44%)	101	45 (45%)	1·7 (1·0–2·8)	1·9 (1·0–3·4)
	p value	..	..	..	..	..	0·40

GP=general practitioner. OR=odds ratio. NA=not applicable.

* Restricted to patients who responded that they had both first sought help from the GP (QWHERE) and that they had visited the GP before being referred (QVISIT1); see appendix for details.

† Patients with prereferral consultation and covariate information.

After excluding 27 participants because of incomplete outcome data, 803 patients had complete information on their symptom onset-to-diagnosis interval, with a median interval of 62 days (IQR 29–153) and a high degree of positive skew across centiles (table 4). Substantial variation was observed in the 50th (ie, median) and 90th centiles of the symptom onset-to-diagnosis interval by cancer site and each sociodemographic variable except ethnic group (table 4). Cancer site was strongly associated with the length of symptom onset-to-diagnosis interval; the majority of this variation was around the 75th to 90th centiles, in which longer symptom onset-to-diagnosis intervals were apparent for unclassified or unspecific neoplasms, soft tissue sarcomas, and bone tumours (appendix). In multivariable quantile regression, significant variation was seen in the 50th (p=0·0005) and 90th (p=0·0034) centiles by sex and cancer site (table 4). The adjusted median symptom onset-to-diagnosis interval for female AYAs was longer than for male AYAs by 24 (95% CI 11–37) days. The longest median symptom onset-to-diagnosis intervals were observed in patients diagnosed with bone tumours (51 [29–73] days longer than for lymphoma) and the shortest in patients diagnosed with leukaemia (33 [17–49] days shorter than for lymphoma). Although no significant variation was seen in the length of the symptom onset-to-diagnosis interval at the 50th centile by employment status, this was the case at the 90th centile (table 4). Patients who were long-term sick had the longest adjusted 90th centile of symptom onset-to-diagnosis interval (222 [90–353] days longer than for those in education).

**Table 4:** Associations of symptom onset-to-diagnosis interval with sociodemographic variables and cancer site

		**Descriptive statistics for all patients with complete symptom onset-to-diagnosis interval**						Multivariable quantile regression estimates (n=789*)	
		N	10th	25th	50th	75th	90th	50th centile (median)	90th centile
								Coefficient (intercept 57·7 days; 95% CI)	Coefficient (intercept 169·2 days; 95% CI)
All patients		803	2	29	62	153	307	NA	NA
Sex									
	Male	439	2	22	58	123	235	1 (ref)	1 (ref)
	Female	364	6	32	84	187	367	24 (11 to 37)	104 (34 to 173)
	p value	..	..	..	..	..	..	0·0005	0·0034
Age at diagnosis (years)									
	12–15	114	1	25	50	100	215	1 (ref)	1 (ref)
	16–18	179	1	17	60	152	322	6 (−7 to 19)	69 (−90 to 229)
	19–24	510	4·5	32	70	166	340	17 (−2 to 35)	84 (−72 to 240)
	p value	..	..	..	..	..	..	0·20	0·57
Index of Multiple Deprivation									
	First quintile (least deprived)	176	3	30	74·5	171·5	365	1 (ref)	1 (ref)
	Second quintile	129	2	30	64	148	275	−6 (−26 to 14)	−17 (−128 to 95)
	Third quintile	155	1	26	56	150	371	−22 (−39 to −6)	47 (−63 to 157)
	Fourth quintile	177	4	32	62	151	278	−7 (−24 to 10)	17 (−93 to 127)
	Fifth quintile (most deprived)	153	4	24	60	148	305	−10 (−33 to 13)	−10 (−117 to 97)
	p value	..	..	..	..	..	..	0·040	0·65
Ethnic group									
	White	706	2	29	62	152	307	1 (ref)	1 (ref)
	Non-white	97	1	29	68	155	338	12 (−14 to 38)	−21 (−115 to 74)
	p value	..	..	..	..	..	..	0·36	0·67
Cancer site									
	Lymphoma	256	5	32	67·5	159·5	338	1 (ref)	1 (ref)
	Germ-cell tumours	153	3	23	57	101	182	−23 (−38 to −7)	−67 (−152 to 19)
	Leukaemia	103	0	15	33	78	161	−33 (−49 to −17)	−65 (−183 to 52)
	Carcinomas	97	1	32	99	188	367	12 (−25 to 49)	−17 (−127 to 94)
	Bone tumours	77	17	53	107	222	400	51 (29 to 73)	101 (−117 to 319)
	Soft tissue sarcomas	43	1	46	78	211	502	14 (−7 to 36)	245 (145 to 345)
	CNS	33	6	30	56	166	342	−1 (−33 to 31)	82 (−484 to 647)
	Melanoma	31	1	8	89	219	259	6 (−57 to 69)	−38 (−123 to 47)
	Unclassified or unspecified	10	6	15	90·5	250	806·5	38 (−161 to 237)	92 (−1958 to 2141)
	p value	..	..	..	..	..	..	<0·0001	<0·0001
Marital status									
	Married, civil partnership, or cohabiting	114	14	33	78	185	383	1 (ref)	1 (ref)
	Single or divorced	688	1	27	62	147·5	296	−12 (−39 to 15)	7 (−79 to 94)
	p value	..	..	..	..	..	..	0·38	0·87
Employment status									
	Education	264	1	22	54	130	227	1 (ref)	1 (ref)
	Working full-time or part-time	251	5	32	66	167	296	7 (−11 to 26)	4 (−72 to 80)
	Other work (apprentice, intern, or voluntary)	17	19	32	100	151	381	35 (−14 to 85)	151 (−300 to 603)
	Not seeking work	121	7	29	63	158	262	−12 (−30 to 6)	−67 (−193 to 59)
	Unemployed	28	1	18	60	125·5	215	2 (−39 to 44)	−36 (−124 to 52)
	Long-term sick	122	6	32	85·5	193	581	20 (−12 to 52)	222 (90 to 353)
	p value	..	..	..	..	..	..	0·25	0·014

NA=not applicable.

* Patients with symptom onset-to-diagnosis and covariate information.

## Discussion

We investigated diagnostic timeliness in AYAs with cancer and examined related variation by sociodemographic characteristic and cancer site. Compared with male AYAs, female AYAs were more likely to have multiple prereferral GP consultations and longer median symptom-onset-to-diagnosis intervals. Further study is needed to understand causes underlying these patterns. There were large variations by cancer site in all aspects of diagnostic timeliness studied. For example, AYAs with melanoma were most likely to wait longer than a month before seeking help about their symptoms, but, by contrast, were the least likely to have multiple prereferral consultations, consistent with the readily identifiable clinical features of this cancer. Patients with lymphoma or bone tumours were most likely to have multiple prereferral consultations, reflecting the often less specific presenting features and the greater diagnostic difficulty of these cancers. The median time from symptom onset to diagnosis was longest for bone tumours and shortest for leukaemia.

Comparison with existing scientific literature is difficult because studies focusing solely on AYAs are rare. This study is the largest one so far examining diagnostic timeliness in AYAs, which was found to be poorer than that reported for children or older adults with cancer.^4^, ^6^, ^7^, ^27^, ^29^ In the present study, among AYAs who consulted with their GP, about one in three had three or more prereferral consultations compared with substantially lower percentages reported previously for chiefly adult English patients with cancer (18–23%).^27^, ^29^

We know of no comparable studies investigating diagnostic intervals across all cancer sites in AYA patients. Most previous studies had small study samples that were restricted to only some cancer sites, or combined data from AYAs with those of children or older adults. A systematic review of 32 studies summarising patient and diagnostic intervals in children and young people with cancer found only one study of 235 patients in the USA reporting exclusively on AYAs.^30^ The proportion of patients in our sample consulting their GP three or more times before a referral (35%) was similar to that reported for the youngest age group (16–24 years) of respondents to the 2010 English Cancer Patient Experience Survey (42%).^27^

Our sample of more than 800 AYAs with high completeness of exposure data allowed us to study associations between diagnostic timeliness outcomes and sociodemographic and cancer site variables, after adjusting for potential confounding through multivariable regression. Survey participants were generally representative of incident cancer cases, although brain tumours and melanoma were slightly under-represented and sarcomas over-represented in our sample (data not shown), which might reflect differences in the nature and frequency of contact of these patient groups with hospital services. For example, the management of melanoma is generally surgical and conducted by skin cancer teams, which are frequently not co-located with AYA services, and surgical centres for brain tumours are not always well linked with AYA services. Patient interviews were done relatively close to the time of diagnosis (on average within 6 months), minimising concerns about potential survivorship bias (whereby patient groups with high risk of mortality post-diagnosis are under-represented among surveyed participants) or recall bias.

Despite substantial efforts, around 50% of patients eligible to participate in this study were not invited to take part because of professional and organisational barriers.^31^ As is common in population surveys, there was a degree of missing data for some outcomes. Although diagnostic timeliness in invited patients and in those analysed might differ, the patterns of variation in the studied outcomes by patient characteristic are unlikely to vary.^32^

Inaccuracies in the recall of first symptom are possible, the effect of which will vary by cancer site. For example, a young person might more easily recall first noticing a definitive skin lesion than symptoms such as fatigue or bone pain, which could partly be normalised by the patient, a family member, or a GP,^33^ and health-care contact might only be triggered after an accumulation of vague symptoms. Some evidence suggests that young people might wait until a threshold is reached, such as symptoms interfering with everyday life or unbearable pain, before seeking care.^33^ Such differences in recall could result in more accurate estimates about the diagnostic timeliness of cancer sites associated with specific symptoms, such as melanoma or male germ-cell cancers, and the potential for greater inaccuracy for cancer sites associated with symptoms of relatively low predictive value. Nevertheless, we observe sufficient evidence for variation by cancer site.

A limitation of our study is that some associations might have appeared significant by chance as a result of testing for multiple exposures. We nonetheless observed strong evidence (p values <0·01) for variation by sex and cancer site with respect to prereferral consultations and the length of the symptom onset-to-diagnosis interval, which minimises, although it does not preclude, concerns about chance associations due to multiple testing.

Although our study is the largest so far to examine diagnostic timeliness in AYAs, the number of categories in certain variables (eg, the number of cancer site groups, and therefore the number of cancer–sex–age–deprivation–ethnicity strata in multivariable models) means that certain estimates of associations have low precision. In such circumstances, focusing on overall patterns of variation between categories rather than category-specific estimates is preferable. Notably, cancer is a heterogeneous disease, and as we describe, diagnostic timeliness varies by cancer site. In our multivariable analysis, estimates for sociodemographic variables are adjusted for the cancer site case-mix in our sample; therefore, they represent an average effect of, for example, sex, on diagnostic timeliness across AYAs with any cancer site. In truth, the association with sex might vary between different cancer sites, but we have not been able to investigate interactions between different exposure variables on the outcomes of interest—eg, whether the greater proportion of three or more prereferral consultations observed in female AYAs is the same across cancer sites or whether it varies between cancers.

Early diagnosis is a global priority in cancer control. Worldwide, 350 000 incident cancer cases per year are estimated in young people aged 15–29 years, and this is increasing.^1^, ^34^ Reducing cancer-related disease burden and improving cancer outcomes in this age group is therefore a priority. However, earlier diagnosis as a potential strategy to improve outcomes has received little or no attention; consequently, little evidence exists currently upon which to base interventions. For the first time, within a group of cancers that are generally difficult to diagnose and rare, we have identified subgroups at higher risk of prolonged intervals to diagnosis. These findings present several opportunities for public health and health-care interventions aimed at shortening diagnostic intervals for AYAs, which can initially target female AYAs and those with symptoms suggestive of melanoma, lymphoma, and bone tumours. Longer time to help-seeking for melanoma symptoms mirrors findings from previous quantitative and qualitative studies in adult patients, which suggest that indicative skin changes are often normalised.^24^, ^33^, ^35^ These data would lend support to awareness campaigns educating AYAs about melanoma risk and symptoms, particularly in view of its increasing incidence. Young people do consult with their GP for a range of reasons (eg, for contraception [in female AYAs] or infections), and these visits present opportunities to educate AYAs about cancer prevention and relevant symptoms. Further research is needed to investigate the psychological, sociological, and circumstantial factors contributing to timely presentation, referral, and diagnosis. The length of the patient interval might be associated with factors such as a lack of awareness of cancer symptoms among AYAs, reassurance by family and friends, inconvenience or competing priorities delaying making or attending GP appointments, or lack of experience of accessing the health-care system. Furthermore, symptoms are often initially attributed to other common illnesses or injuries by both patients and their GPs, particularly in view of the low risk of cancer in this age group.

Female AYAs and AYAs with lymphoma or bone tumours were more likely to experience multiple GP consultations before being referred for suspicion of cancer. This finding mirrors similar differences reported in older patients with cancer.^27^ For young people, symptoms initially might be falsely attributed to other common illnesses in young adults, such as musculoskeletal (including sports-related) or gynaecological complaints.^36^ This is supported by our findings that patients with melanoma and germ-cell tumours (including testicular cancer), which are typically associated with fairly specific symptoms that are easy to examine, were some of the least likely to consult multiple times. Although the prevalence of tumours among AYAs is relatively low,^14^, ^23^ previous research in primary care records has estimated that the presence of head lump mass or neck or lymph node swelling increases the likelihood of lymphoma by 200–400 times, and any lump, mass, or swelling increases the likelihood of bone tumours or soft tissue sarcomas by around 80 times.^14^ Therefore, there is a need, not only for rapid referral for tests either to a secondary care clinician or to diagnostic service following such symptoms in AYAs, as per clinical guidelines,^37^ but also for new diagnostic technologies to aid GP decision making.

Although certain subgroups of AYAs are at increased risk of prolonged time intervals to diagnosis, the related effect on clinical and patient experience outcomes is yet to be fully understood. A systematic review^20^ of the associations between time to diagnosis or treatment and clinical outcomes encompassed patients aged 0–24 years as a single age group, without disaggregating the data by age. It reported mixed findings regarding stage at diagnosis and survival depending on cancer site and no studies investigating associations for patients with either of the two most common cancers in AYAs: lymphoma and germ-cell tumours. In addition to clinical and patient experience improvements, the societal gains associated with early diagnosis are likely to be substantial in view of the life-years lost or at risk in people diagnosed with cancer at an early age.

In summary, we have assessed patient-reported data on diagnostic timeliness in a large representative multicentre cohort of AYAs with cancer in England. We have identified subgroups at greater risk of prolonged intervals from symptom onset to presentation and diagnosis, in whom further research and early diagnosis interventions might be additionally targeted to maximise their effectiveness (ie, female AYAs and AYAs with symptoms or signs of melanoma, lymphoma, or bone tumours). Despite its rarity, early detection of cancer in AYAs warrants prioritisation because of the societal and economic gains that can result from improved survival and psychosocial outcomes in this group. However, because of the difficulty of diagnosing cancer in AYAs, innovations in diagnostic technologies (including point-of-care rule-out tests or algorithms to be used in primary care) also need to be developed and assessed.
