# Supplementary material for: Diagnostic timeliness in adolescents and young adults with cancer: a cross-sectional analysis of the BRIGHTLIGHT cohort
Source: Lancet Child Adolesc Health. 2018 Mar;2(3):180–90. doi: 10.1016/S2352-4642(18)30004-X (PMC5824669; doi:10.1016/S2352-4642(18)30004-X)
Supplement: Supplementary appendix [file mmc1.pdf]

THE LANCET
Child & Adolescent Health

Supplementary appendix

This appendix formed part of the original submission and has been peer reviewed. We post it as supplied by the authors.

Supplement to: Herbert A, Lyratzopoulos G, Whelan J, et al. Diagnostic timeliness in adolescents and young adults with cancer: a cross-sectional analysis of the BRIGHTLIGHT cohort. *Lancet Child Adolesc Health* 2018; published online Jan 29. http://dx.doi.org/10.1016/S2352-4642(18)30004-X.

Diagnostic timeliness in adolescents and young adults with cancer: Cross-sectional findings from the BRIGHTLIGHT cohort

Annie Herbert; Georgios Lyratzopoulos; Jeremy Whelan; Rachel M Taylor; Julie Barber; Faith Gibson; Lorna Fern

Supplementary material

Table S1: Survey questions* used to define intervals and number of pre-referral consultations

Figure S1: Flow chart of BRIGHTLIGHT participants

Figure S2: Quantile plot for total interval, by cancer site (n=803)

Table S2: STROBE checklist

Text S1: Study protocol (June 2016)

Text S2: Text S2: Associations between lag time, date of diagnosis as recorded by the Cancer Registry and the time of survey/interview completion, and the related 3 measures of diagnostic timeliness

Table S3: Deviation between study protocol and final study

Table S1: Survey questions* used to define intervals and number of pre-referral consultations

Synoptic form	Question verbatim (variable name)	Response options	How variable used in current analysis
Place of first presentation	"Looking at this list, please can you tell me where you first went to discuss your symptom(s)?" (QWHERE)	Single answer option: GP practice [to see GP or Practice Nurse], A&E [Casualty], Pharmacist, Physiotherapist, Optician, Dentist, School nurse, Other [please state], Don't know/can't remember	Responses used to restrict our analysis to a sub-sample of patients who first sought help from a GP or A&E, in order to define the patient interval, and to exclude patients with contradictory responses when defining the number of pre-referral GP consultations (see QVISIT below for detailed explanation).
Month/year of symptom(s) onset	"When did you first think something might be wrong with your body? Please tell me the month and year when you first thought this?" (QDIAG)	Must enter a month between January and December and a year between 2011 and 2014. If respondent unsure, asked for best guess.	Response used to define the start of the total interval. We assumed that the date of noticing first symptom was the mid-point of the month (i.e. the 14th).
Time from symptom onset to first presentation	"From the time when you first noticed a symptom of cancer how long was it before you saw <insert from QWHERE>?" (QSEEKH)	Single answer option: Under 1 week, 1 week up to 2 weeks, Over 2 weeks up to 4 weeks, Over 1 month up to 3 months, Over 3 months up to 6 months, Over 6 months up to 12 months	Responses used to define the patient interval.
Care facility where pre-referral care was sough (e.g. GP, A&E)	"Between the time you first noticed your symptom(s) and being referred for medical test or investigation which, if any, of the places listed on this card did you visit?" (QVISIT)	Multiple answer option: GP practice, A&E [Casualty], Another hospital ward/department (i.e. not A&E), Walk-in centre, Polyclinic, None of these, Don't know/can't remember, Other please state	Responses used to more robustly define the number of patients with pre-referral consultations, i.e. to exclude patients who in response to QWHERE they indicated that they had first sought help from the GP, but in response to QVISIT1 indicated that they had not visited the GP before being referred, n = 125).
Number of pre-referral consultations at given care facility	"How many times did you go <pull through response from QVISIT> before you were diagnosed?" (QNUMVISIT1)	Enter number between 1 and 40; or 'don't know/can't remember'	Response used to define number of pre-referral GP consultations (after excluding contradictory responses – see row above).

*Available here: https://xip.uclb.com/i/healthcare_tools/brightlight_wave1.html, under licence.

Figure S1: Flow chart of BRIGHTLIGHT participants

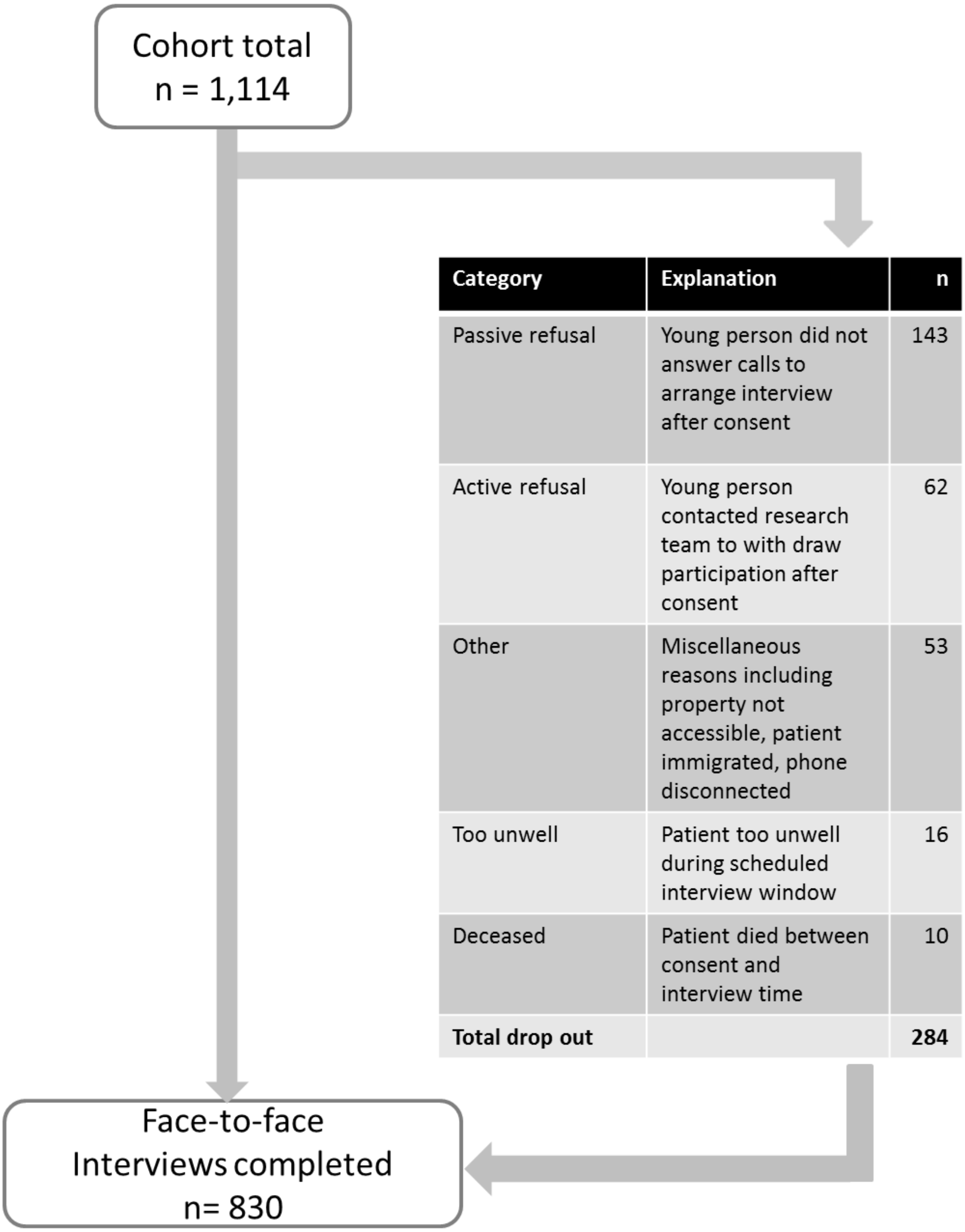

Category	Explanation	n
Passive refusal	Young person did not answer calls to arrange interview after consent	143
Active refusal	Young person contacted research team to with draw participation after consent	62
Other	Miscellaneous reasons including property not accessible, patient immigrated, phone disconnected	53
Too unwell	Patient too unwell during scheduled interview window	16
Deceased	Patient died between consent and interview time	10
**Total drop out**		**284**

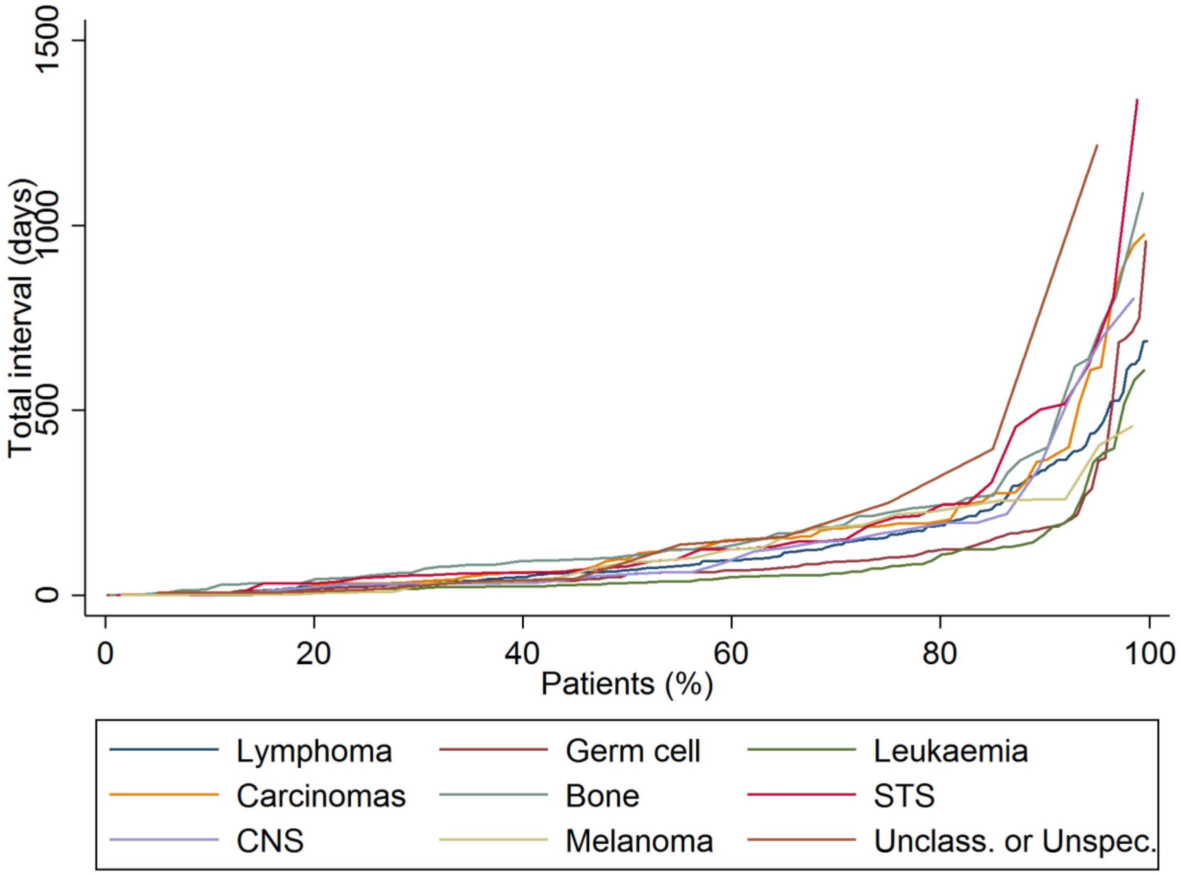

Bone = Osseous and chondromatous neoplasms, Ewing’s tumour and other neoplasms of bone; CNS = Central nervous system and other intracranial and intraspinal neoplasms; Germ cell = Germ cell and trophoblastic neoplasms; STS = Soft Tissue Sarcomas; Unclass. or Unspec. = Unclassified or Unspecified

Figure S2: Quantile plot for total interval, by cancer site (n=803)

Table S2: STROBE Statement—Checklist of items that should be included in reports of *cross-sectional studies*

	Item No	Recommendation
**Title and abstract**	1	(*a*) Indicate the study's design with a commonly used term in the title or the abstract<br>Title: Diagnostic timeliness in adolescents and young adults with cancer: Cross-sectional findings from the BRIGHTLIGHT cohort
		(*b*) Provide in the abstract an informative and balanced summary of what was done and what was found<br>*Lines 38-53* (Methods & Findings).
**Introduction**		
Background/rationale	2	Explain the scientific background and rationale for the investigation being reported<br>*Lines 63-65:* Cancer is the leading cause of disease death for adolescents and young adults (AYA) in the developed world.[1] Improvements in survival for this population have lagged behind both children and older adults<br><br>*Lines 67-74:* Some evidence suggests that young people experience a longer time from symptom onset to diagnosis compared to children and older adults.[4, 6-9] However, the evidence is difficult to interpret and inconclusive as comparable studies are rare. Prolonged intervals to diagnosis can adversely affect clinical outcomes, decrease confidence of patients and parents in their doctors and are associated with a poorer experience of subsequent cancer care.[10, 11] Despite this, diagnostic timeliness in AYA is not well quantified and the identification of high-risk groups remains elusive.<br><br>*Lines 88-91:*<br>We aimed to examine diagnostic timeliness of cancer in a cohort of young people, to identify factors associated with longer diagnostic journeys and produce evidence to inform interventions supporting earlier diagnosis and improvement of subsequent outcomes.
Objectives	3	State specific objectives, including any prespecified hypotheses<br>*Lines 88-91:*<br>We aimed to examine diagnostic timeliness of cancer in a cohort of young people, to identify factors associated with longer diagnostic journeys and produce evidence to inform interventions supporting earlier diagnosis and improvement of subsequent outcomes.
**Methods**		
Study design	4	Present key elements of study design early in the paper<br>*Lines 94-97: "*We cross-sectionally analysed survey data from the BRIGHTLIGHT cohort, linked to 'Case Report Form' (CRF) data (completed by recruiting clinical treatment teams) and cancer registration data (received from Public Health England National Cancer Registration Analysis Service [PHE NCRAS])."
Setting	5	Describe the setting, locations, and relevant dates, including periods of recruitment, exposure, follow-up, and data collection<br>*Lines 99-107:* BRIGHTLIGHT is a programme of research evaluating specialist care for young people with cancer in England.[16] Treatment teams identified participants as patients aged 13-24 with any new primary cancer diagnosis during September 2012-April 2015. Exclusion criteria were: unable to complete the survey; unable to give consent; facing imminent death or serving a custodial sentence due to the impracticalities of obtaining consent. BRIGHTLIGHT recruited 1,114 participants within four months of cancer diagnosis from 96 National Health Service Trusts hospitals across England which deliver free universal healthcare to all patients in their geographical catchment. Of those recruited, 830 completed a face-to-face interview.
Participants	6	(*a*) Give the eligibility criteria, and the sources and methods of selection of participants<br>*Lines 100-106:* Treatment teams identified participants as patients aged 13-24 with any new primary cancer diagnosis during September 2012-April 2015. Exclusion criteria were: unable to complete the survey; unable to give consent; facing imminent death or serving a custodial sentence due to the impracticalities of obtaining consent. BRIGHTLIGHT recruited 1,114 participants within four months of cancer diagnosis from 96 National Health Service Trusts hospitals across England which deliver free universal healthcare to all patients in their geographical catchment. Of those recruited, 830 completed a face-to-face interview.
Variables	7	Clearly define all outcomes, exposures, predictors, potential confounders, and effect modifiers. Give diagnostic criteria, if applicable.<br>- Outcomes described in standalone section lines 131-135, and derivation described in Supplementary Table S1 and Figure 1.<br>- Exposures described in standalone section (lines 136-145)<br>- Potential confounders, lines 164-165: "We additionally fitted multivariable regression models, including all exposures, to account for potential confounding of crude associations by other variables."<br>- Effect modifiers, lines 171-172: "<br>We did not study interactions between exposures, as we deemed the sample size was not large enough to enable such informative analysis.
Data sources/ measurement	8*	For each variable of interest, give sources of data and details of methods of assessment (measurement). Describe comparability of assessment methods if there is more than one group<br>Sources of data and derivation of variables described in Supplementary Table S1 and Figure 1. Comparability of assessment methods not applicable.
Bias	9	Describe any efforts to address potential sources of bias<br>Footnote to Figure 1: "125 patients responded that they had first sought help from their GP (QWHERE) but did not respond that they had visited their GP pre-referral (QVISIT). These patients

		were excluded” [from analyses of the outcome 3+ consultations, to be sure that this outcome represented 3+ GP consultations]
Study size	10	Explain how the study size was arrived at<br>*Lines 128-129: “*As the current study comprised secondary data analyses, no sample size calculations were conducted.”
Quantitative variables	11	Explain how quantitative variables were handled in the analyses. If applicable, describe which groupings were chosen and why<br>Groupings of exposure variables are described and justified in lines 137-155.<br><br>*Lines 159-160:* ‘We also described the numbers and proportions of patients with 3+ pre-referral GP consultations, as this is associated with the length of primary care interval’<br>*Lines 174-184*: For our two categorical outcomes (i.e. patient interval, and 3+ pre-referral GP consultations), we fitted multivariable logistic regression models. We re-categorised the patient interval as a binary variable (<1 month vs. 1 month+) for analytical and interpretational convenience, informed by prior relevant studies in adult patients.[23, 24] ‘We opted for a one month cut-off, deeming this long enough to be clinically important, and taking into consideration that public health education campaigns about awareness of cancer symptoms typically use a cut-off of 3 weeks for chronic symptoms.’ We treated the number of pre-referral consultations as a binary outcome (<3 vs. 3+), consistent with public reporting conventions of the National Cancer Patient Experience Survey,25 and given that some second appointments could reflect the need to review the findings of investigations ordered at initial consultation.[26]
Statistical methods	12	(*a*) Describe all statistical methods, including those used to control for confounding<br>*Described in a standalone section (lines 156-199).*
		(*b*) Describe any methods used to examine subgroups and interactions<br>*Lines 171-173:* “We did not study interactions between exposures, as the study was not adequately powered to do so.”
		(*c*) Explain how missing data were addressed<br>*Lines 206-208: “*Information was complete for gender, age, and ethnicity, with low levels of missing-ness”<br>*Lines 321-326:* As common in population surveys, there was a degree of missing data for some outcomes, e.g. exclusion of 125 patients (15% of sample) from analysis of 3+ consultations. Further, while on average diagnostic timeliness in invited patients and those in the analysis sample may differ, it is generally accepted in survey research that the patterns of variation in the studied outcomes by patient characteristics are unlikely to differ.
		(*d*) If applicable, describe analytical methods taking account of sampling strategy<br>*Not applicable.*
		(*e*) Describe any sensitivity analyses<br>*None carried out.*
**Results**		
Participants	13*	(a) Report numbers of individuals at each stage of study—eg numbers potentially eligible, examined for eligibility, confirmed eligible, included in the study, completing follow-up, and analysed<br>*Lines 176-177:* Information was analysed for a total of 830 patients (55% male, whose median age was 20 years, (interquartile range 17 to 22); Table 1).<br><br>*Lines 103-112*: BRIGHTLIGHT recruited 1,114 participants within four months of cancer diagnosis from 96 National Health Service Trusts hospitals across England which deliver free universal healthcare to all patients in their geographical catchment. Of those recruited, 830 completed a face-to-face interview within five months of diagnosis. Reasons for study drop out between consent and interview included early death, refusal, and being too ill to take part; these participants were not atypical to those who remained in the study (Supplementary Figure S1). A full description of the BRIGHTLIGHT cohort will be reported separately. Once lLocal treatment teams gained consent from young people agreed to participate in the study, and then young people were contacted by the survey provider (Ipsos MORI) to undertake a structured interview.<br><br>Numbers analysed are described within Tables 2-4, and in titles of Figure 2 and Supplementary Figure S1: Flow chart of BRIGHTLIGHT participants
		(b) Give reasons for non-participation at each stage<br>*Lines 107-110* Reasons for study drop out between consent and interview included early death, refusal, and being too ill, see figure S1 *Flow chart of BRIGHTLIGHT participants*
		(c) Consider use of a flow diagram<br>In ‘Methods’, we reference the BRIGHTLIGHT website which provides links further describing derivation of the cohort. see figure S1 Flow chart of BRIGHTLIGHT participants
Descriptive data	14*	(a) Give characteristics of study participants (eg demographic, clinical, social) and information on exposures and potential confounders<br>These are described in Table 1.
		(b) Indicate number of participants with missing data for each variable of interest<br>These are described in Table 1, and as footnotes to Tables 2-4.
Outcome data	15*	Report numbers of outcome events or summary measures<br>Reported in Tables 2-5.
Main results	16	(*a*) Give unadjusted estimates and, if applicable, confounder-adjusted estimates and their precision (eg, 95% confidence interval). Make clear which confounders were adjusted for and why they were included.<br>Both unadjusted and adjusted odds ratios or coefficients are provided within Tables 2-4.

		As described in lines 163-165: "We additionally fitted multivariable regression models, including all exposures, to account for potential confounding of crude associations by other variables."
		(*b*) Report category boundaries when continuous variables were categorized<br>Not applicable.
		(*c*) If relevant, consider translating estimates of relative risk into absolute risk for a meaningful time period<br>Not applicable (period of recruitment/data collection was less than three years).
Other analyses	17	Report other analyses done—eg analyses of subgroups and interactions, and sensitivity analyses<br>Not applicable.
**Discussion**		
Key results	18	Summarise key results with reference to study objectives<br>*Lines 283-293* We examined diagnostic timeliness and related it to variation by sociodemographic characteristics and cancer site. Compared to males, females were more likely to have multiple pre-referral GP consultations and the longest average symptom-onset-to-diagnosis interval. Further study is needed into causes underlying these patterns. There was large variation by cancer site in all aspects of diagnostic timeliness studied. AYA with melanoma were most likely to wait over a month before seeking help about their symptoms, but were the least likely to have multiple pre-referral consultations, consistent with the readily identifiable clinical features of this cancer. Patients with lymphoma or bone tumours were most likely to have multiple pre-referral consultations, reflecting the often less-specific presenting features. The average time from first symptom to diagnosis was longest for bone tumours and shortest for leukaemia
Limitations	19	Discuss limitations of the study, taking into account sources of potential bias or imprecision. Discuss both direction and magnitude of any potential bias.<br>Limitations discussed in lines 312-358. Sources of potential bias: missing data, inaccuracy of recall of first symptom. Implications discussed in lines 282-285:<br><br>*Sources of potential bias: missing data, inaccuracy of recall of first symptom. Implications discussed in lines 323-3265*: while on average diagnostic timeliness in invited patients and those in the analysis sample may differ , it is generally accepted in survey research that the patterns of variation in the studied outcomes by patient characteristics are unlikely to differ<br>*And lines 333-338* Such differences in recall could result in more accurate estimates about the diagnostic timeliness of cancer sites associated with specific symptoms, such as melanoma or male germ cell cancers, and the potential for inaccuracy for cancer sites associated with symptoms of relatively low predictive value. Nevertheless, we observe sufficient evidence for variation for some of these latter sites (leukaemia, germ cell tumours, and bone tumours), although these associations may have been under-estimated.
Interpretation	20	Give a cautious overall interpretation of results considering objectives, limitations, multiplicity of analyses, results from similar studies, and other relevant evidence<br>*Lines 315-319* Survey participants were generally representative of incident cancer cases, and patient interviews were carried out relatively close to the time of diagnosis (on average within six months), minimising concerns about potential survivorship bias (whereby patient groups with high risk of mortality post-diagnosis are under-represented among surveyed participants), or recall bias.<br><br>*Lines 323-326: "...*Further, while on average diagnostic timeliness in invited patients and those in the analysis sample may differ , it is generally accepted in survey research that the patterns of variation in the studied outcomes by patient characteristics are unlikely to differ.[26]"<br><br>*Lines 296-298:* Diagnostic timeliness of AYA in this study was poorer compared to that reported for children or older adults with cancer.<br><br>*Lines 345-350: "* Despite the fact that our sample size is the largest study to date of patient-reported diagnostic timeliness for AYA, the number of categories in certain variables (e.g. the number of cancer site groups, and therefore the number of cancer-gender-age-deprivation-ethnicity strata in multivariable models) means that certain estimates of associations lack precision. In such circumstances focusing on overall patterns of variation between categories rather than category-specific estimates is preferable
Generalisability	21	Discuss the generalisability (external validity) of the study results<br>*Line 315:* "Survey participants were generally representative of incident cancer cases"
**Other information**		
Funding	22	Give the source of funding and the role of the funders for the present study and, if applicable, for the original study on which the present article is based<br>*Lines 435-440: "*This paper presents independent research funded by the National Institute for Health Research (NIHR) under its Programme Grants for Applied Research Programme (Grant Reference Number RP-PG-1209-10013). The views expressed are those of the author(s) and not necessarily those of the NHS, the NIHR or the Department of Health. LF is funded by Teenage Cancer Trust. GL is supported by a Cancer Research UK Advanced Clinician Scientist Fellowship Award (Reference A18180)."

*Give information separately for exposed and unexposed groups.

Note: An Explanation and Elaboration article discusses each checklist item and gives methodological background and published examples of transparent reporting. The STROBE checklist is best used in conjunction with this article (freely

available on the Web sites of PLoS Medicine at http://www.plosmedicine.org/, Annals of Internal Medicine at http://www.annals.org/, and Epidemiology at http://www.epidem.com/). Information on the STROBE Initiative is available at www.strobe-statement.org.

Text S1: Study protocol (2nd June 2016)

I. Full Title: Variation in pre-diagnostic intervals for teenagers and young adult cancer patients: findings from the BRIGHLIGHT survey

II. Core Data: Brightlight survey data, linked to case report forms (CRFs).

III. Background

There is evidence that there is variation in the length of different diagnostic intervals (from symptom to presentation ['patient interval'], from presentation to referral ['primary care interval'], from referral to diagnosis ['system interval']) for adult cancer patients (1), according to socio-demographic characteristics such as age and sex, and type of cancer (2-4). However, this variation has not yet been characterised in children and young people. The distribution of cancer types in this population is different to that in adults; it is likely that so is the variation in lengths of diagnostic intervals. We propose to explore variation in lengths of diagnostic intervals in children and young people by socio-demographic characteristics and types of cancer, to highlight any missed opportunities for a more timely diagnosis.

IV. Main Question(s)

What is the variation in pre-diagnostic intervals for teenagers and young adults? How much of this variation is accounted for by socio-demographic factors?

V. Data to be used

Dependent variable: Patient-, Primary Care- and system-, intervals (derived from survey variables QDIAG, QSEEKH, QWAIT3, and histological date of diagnosis [CRF])

Independent variable and control variables: sex (CRF), age at diagnosis (CRF), ethnicity (QETHNIC), socioeconomic status (Index of Multiple Deprivation from CRF), marital status (QMARSTAT; for those 16y+), employment status (QEMPLOY1; for those 16y+), and cancer site (QCANCER).

VI. Analytic plan

For each combination of socio-demographic characteristics and type of cancer, we will present descriptive statistics of the patient, primary care and system intervals (median and other centiles). We will explore the association of these independent variables with each outcome by fitting quantile regression models (which will account for the likely positive skew of outcomes).

References

1. Lyratzopoulos G. Markers and measures of timeliness of cancer diagnosis after symptom onset: a conceptual framework and its implications. Cancer epidemiology. 2014;38(3):211-3.
2. Neal RD, Din NU, Hamilton W, Ukoumunne OC, Carter B, Stapley S, et al. Comparison of cancer diagnostic intervals before and after implementation of NICE guidelines: analysis of data from the UK General Practice Research Database. British journal of cancer. 2014;110(3):584-92.
3. Lyratzopoulos G, Abel GA, McPhail S, Neal RD, Rubin GP. Measures of promptness of cancer diagnosis in primary care: secondary analysis of national audit data on patients with 18 common and rarer cancers. British journal of cancer. 2013;108(3):686-90.
4. Lyratzopoulos G, Neal RD, Barbiere JM, Rubin GP, Abel GA. Variation in number of general practitioner consultations before hospital referral for cancer: findings from the 2010 National Cancer Patient Experience Survey in England. The lancet oncology. 2012;13(4):353-65.

Text S2: Associations between lag time, date of diagnosis as recorded by the Cancer Registry and the time of survey/interview completion, and the related 3 measures of diagnostic timeliness

We tested whether longer time from diagnosis to interview date is associated with shorter/longer Patient Interval or symptom onset to diagnosis interval; or greater / lower % of 3+ pre-referral consultations. Associations between 'lag time' between the date of diagnosis as recorded by the Cancer Registry and the time of survey/interview completion, and the related 3 measures of diagnostic timeliness in our study were examined. We have examined this association both crudely and after adjustment for age group, sex, deprivation and cancer site.

There was no evidence to support an association between lag time and odds of the patient interval being greater than 2 weeks; 4 weeks; or 3 months table below; the 2 rows represent different logistic regression models):

Patient interval	OR (95% CI)	P-value
>4 weeks (crude)	1.0 (1.0–1.0)	0.726
>4 weeks (adjusted)	1.0 (1.0–1.0)	0.661

Similar results were obtained when parameterising the Patient Interval using binary cut-offs of 2 weeks or 3 months. Similarly, across the range of values of the symptom onset to diagnosis interval, there was no evidence for such an association with lag time (p=0.993 crude, p=0.744). Lastly, we examined the association between lag time and 3+ pre-referral consultations, and again found no evidence for an association (crude p=0.771; adjusted p=0.747).

.

Table S3: Deviation between study protocol and final study

Protocol	Final study	Justification
"Core Data: Brightlight survey data, linked to case report forms (CRFs)."	Core Data: Brightlight survey data, linked to case report forms (CRFs) and cancer registration data.	Between the protocol being written and study being carried out, linked cancer registration data became available, which would likely provide a more accurate date of diagnosis and definition of cancer site than survey data.
"Dependent variable: Patient-, Primary Care- and system-, intervals (derived from survey variables QDIAG, QSEEKH, QWAIT3, and histological date of diagnosis [CRF])"	Dependent variable: Patient interval, 3+ pre-referral GP consultations, and total interval (derived from survey variables QWHERE, QDIAG, QSEEKH, QVISIT, QNUMVISIT1, QDIAG_MONTH, QDIAG YEAR and cancer registry diagnosis date)	After exploration of the survey data, linked cancer registry diagnosis date became available, and it became clear that: QWHERE was needed to derived patient interval (to determine that patient had first sought help from either the GP or A&E); primary care interval could not be derived from patient interval and QWAIT3 (time from first noticing symptom to first being referred) as planned, as the categorical outcomes for patient interval and QWAIT3 could not easily be subtracted from one another; QVISIT and QNUMVISIT1 provided the outcome 3+ pre-referral GP consultations, which is itself a proxy for primary care interval;[1] QDIAG_MONTH, QDIAG_YEAR and cancer registry diagnosis date could be used to derive the total interval.
"Independent variable and control variables: …cancer site (QCANCER)."	Cancer site was determined by the cancer registry data.	Between the protocol being written and study being carried out, linked cancer registration data became available, which would likely provide a more accurate definition of cancer site than survey data.
"We will explore the association of these independent variables with each outcome by fitting quantile regression models (which will account for the likely positive skew of outcomes)."	Additional to quantile regression models, we also fitted logistic regression models.	Patient interval and 3+ pre-referral GP consultations were only available as categorical outcomes.

1. Lyratzopoulos G, Abel GA, McPhail S, Neal RD, Rubin GP. Measures of promptness of cancer diagnosis in primary care: secondary analysis of national audit data on patients with 18 common and rarer cancers. *Br J Cancer* 2013; **108**(3): 686-90.
